# Model Reference Predictive Adaptive Control for Large-Scale Soft Robots

**DOI:** 10.3389/frobt.2020.558027

**Published:** 2020-10-05

**Authors:** Phillip Hyatt, Curtis C. Johnson, Marc D. Killpack

**Affiliations:** Robotics and Dynamics Lab, Department of Mechanical Engineering, Brigham Young University, Provo, UT, United States

**Keywords:** model predictive control, adaptive control, continuum robot, dynamic modeling, MRAC, parameter mismatch, structure mismatch, soft robot

## Abstract

Past work has shown model predictive control (MPC) to be an effective strategy for controlling continuum joint soft robots using basic lumped-parameter models. However, the inaccuracies of these models often mean that an integral control scheme must be combined with MPC. In this paper we present a novel dynamic model formulation for continuum joint soft robots that is more accurate than previous models yet remains tractable for fast MPC. This model is based on a piecewise constant curvature (PCC) assumption and a relatively new kinematic representation that allows for computationally efficient state prediction. However, due to the difficulty in determining model parameters (e.g., inertias, damping, and spring effects) as well as effects common in continuum joint soft robots (hysteresis, complex pressure dynamics, etc.), we submit that regardless of the model selected, most model-based controllers of continuum joint soft robots would benefit from online model adaptation. Therefore, in this paper we also present a form of adaptive model predictive control based on model reference adaptive control (MRAC). We show that like MRAC, model reference predictive adaptive control (MRPAC) is able to compensate for “parameter mismatch" such as unknown inertia values. Our experiments also show that like MPC, MRPAC is robust to “structure mismatch” such as unmodeled disturbance forces not represented in the form of the adaptive regressor model. Experiments in simulation and hardware show that MRPAC outperforms individual MPC and MRAC.

## 1. Introduction

Large-scale soft robots hold promise as platforms that are safe for human and delicate environments, and are able to accomplish tasks for which rigid robots are ill-suited. Some tasks for which large-scale soft robots are uniquely capable include whole-arm wiping tasks, reaching through unmodeled cluttered environments, and any task where incidental unmodeled contact is likely or desirable. Continuum joint soft robots have specifically been modeled after examples in nature that excel at these types of tasks (anteaters, octopi, elephants, etc.).

One major obstacle to the use of continuum joint soft robots is the lack of accurate models to enable model-based control. Because flexible continuum joints are not necessarily constrained to rotate about a single well-defined axis, even the kinematic modeling of these robots is relatively complex when compared to rigid robots. The rigid-body dynamics equation that govern the motion of traditional robots are further complicated in pneumatically-actuated continuum joint soft robots by pressure dynamics, energy storage and dissipation in the joints, as well as buckling in some load cases. These factors make the accurate modeling and model-based control of continuum joint soft robots very difficult.

In this work we present a novel dynamic model of a continuum joint robot that can be evaluated fast enough for real-time model predictive control (MPC). This novel dynamic model is in fact a small extension of well-established dynamic model of continuum joint robots based on piecewise constant curvature (PCC) approximations, and a relatively new choice of configuration variables. While only derived for a one joint robot (two degree of freedom), the ideas in this paper are extensible to continuum joint robots with multiple actuated joints.

We also present a form of adaptive MPC that can update our model in order to improve dynamic performance and eliminate steady state error. The adaptive law and much of the theoretical basis for this controller are derived from model reference adaptive control (MRAC) techniques.

The structure of this paper is as follows: section 2 presents the state of the art in continuum soft robot modeling and control, as well as the hardware, models and methods specific to this work; section 3 explains our hypotheses about the new model and proposed controller as well as the design of the experiments performed; section 4 shows the results of the experiments performed and discusses their importance; section 5 discusses the importance of the presented work to the field and provides suggestions for future work.

### 1.1. Related Work

There is a significant body of work relative to accurately modeling the kinematics and dynamics of soft robots. In Renda et al. (2012) and Thuruthel et al. (2016) the continuum joint is modeled using Cosserat-beam theory. In Kang et al. (2011) and Khalil et al. (2007) methods based on recursive Newton-Euler approaches are used, while in Tatlicioglu et al. (2007) and Godage et al. (2011) dynamic equations are derived using Lagrangian mechanics. In Zheng et al. (2012) and Giri and Walker (2011) lumped parameter models are derived by dividing the continuum joint into a number of finite length sections. The trade-off between accuracy and computational complexity in these methods can be seen by varying the number of the finite sections. The authors of Walker (2013) provide a more comprehensive review of dynamic models for soft and continuum joint robots. Notably, there has also been work to show that learned models can represent soft robot dynamics as in Thuruthel et al. (2017).

In Mochiyama and Suzuki (2002) and Mochiyama and Suzuki (2003) the authors derive the dynamic equations of a continuum arm by integrating over infinitesimal disks and using the method of Lagrange. No assumptions of constant curvature are made. These works are similar to the modeling efforts presented in this paper, the main differences being our choice of generalized coordinates and our assumption of constant curvature. These two differences allow us to derive closed-form analytical expressions for the terms in our equations of motion such as the mass and Coriolis matrices.

In Falkenhahn et al. (2014) and Falkenhahn et al. (2015) the authors derive simpler models based on the PCC assumption. However, they neglect generalized forces caused by rotational inertias. They also model the mass of each PCC section as being concentrated at a point that is fixed in some coordinate frame. In Della Santina et al. (2020b), the authors derive a similar PCC-based model (also neglecting rotational inertia) and then match it to a dynamically equivalent rigid body model. Because the mass and inertia of the joints used in our work are non-negligible, we model the mass as distributed uniformly throughout infinitesimal disks and the center of mass of each joint is calculated analytically assuming uniform density. This approach yields closed-form equations of motion for the continuum joint while more accurately representing the dynamics by including the effects of rotational inertia. This approach also illustrates to a greater extent the effect of dynamic models that include rotational inertia on the performance of highly underdamped systems when compared to the work found in Della Santina et al. (2020b).

Control strategies for soft robots vary from open-loop control such as in Shepherd et al. (2011) and Tolley et al. (2014) to Reinforcement Learning (Zhang et al., 2017) to model predictive control (Best et al., 2016). In Hyatt et al. (2019) and Hyatt and Killpack (2020) the authors demonstrate the performance of MPC on the same joints used for this work. These implementations of MPC used a learned model of the dynamics based on a less-accurate representation of the continuum joint dynamics. The model inaccuracy that resulted in less aggressive control in that work prompted the development of the more accurate model and adaptive control techniques presented in this paper.

Given a dynamic model of the correct form, the nature of soft robots is still such that certain parameters of that model may be difficult to estimate. In terms of adaptive control for soft robots, the most similar to our work is Trumić et al. (2020), where they use a similar formulation of MRAC (although with a different dynamic model and no optimal control law). Although not common in soft robotics, combining MPC and adaptive control is beginning to be an established control strategy where the strengths of MPC are combined with a variety of adaptive control schemes (see Adetola et al., 2009; Kim, 2010; Chowdhary et al., 2013; Bujarbaruah et al., 2018; Pereida and Schoellig, 2018; Abdollahi and Chowdhary, 2019; Zhang and Shi, 2020). The method developed in this paper is a unique form of adaptive MPC that borrows ideas from model reference adaptive control (MRAC) for robot manipulators (Slotine and Li, 1987). Specifically, our work can be considered an extension to the adaptive MPC presented in Terry et al. (2019). The main extensions are an adaptive law formulated specifically for robot manipulators and a regressor based on a more accurate continuum joint dynamic model. These extensions allow greater flexibility to adapt both the parameters and structure of the model.

## 2. Materials and Methods

### 2.1. Robot Platform Description and Modeling

**Figure 1:**
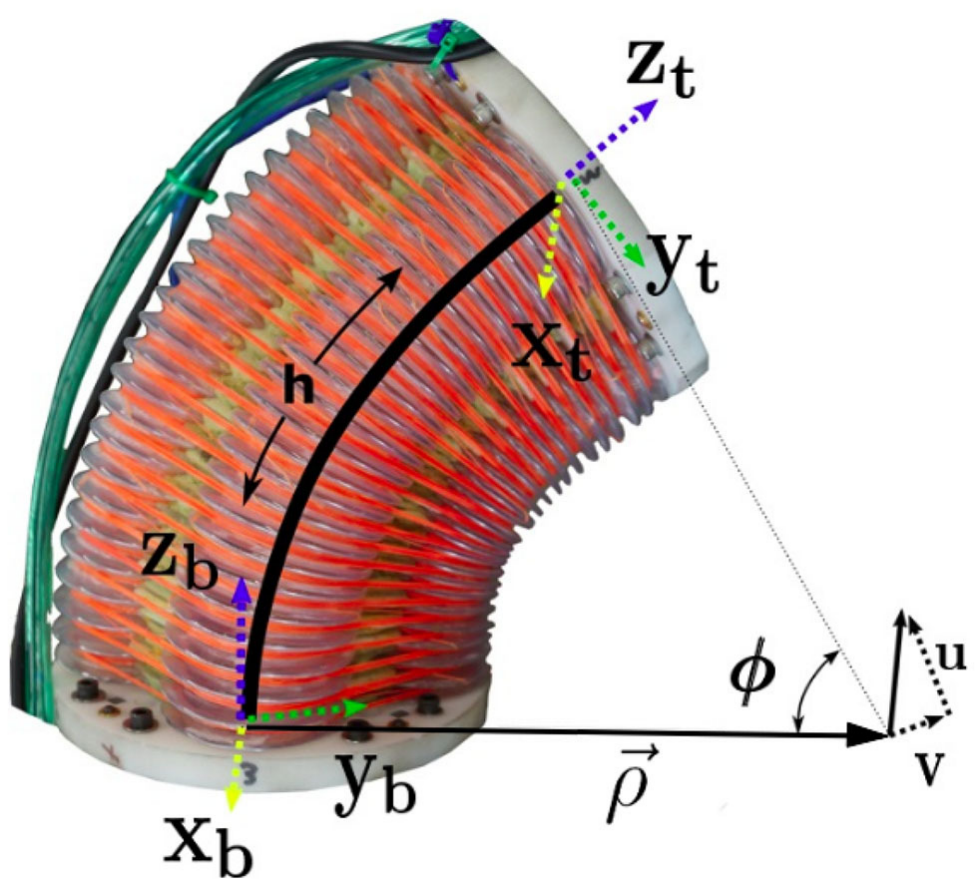
A continuum joint robot such as the one used for this work. The variables $\vec{\rho }$, ϕ, *u*, *v*, and *h* are labeled for reference.

The robot used for this work is composed of a continuum joint such as the one seen in Figure 1. These joints are made of four separate pressure-controlled chambers surrounding a relatively inextensible central cable. The two antagonistically placed pairs of pressure chambers allow the joint to bend about two axes. We choose to model the kinematics of this joint using arcs of constant curvature. Each arc, which traces out the path in space occupied by the inextensible spine, can be defined using three variables as described in Allen et al. (2020) (see similar derivation in Della Santina et al., 2020a). These variables are the length of the in-extensible spine (*h*) and two components of the axis-angle vector that describes the rotation from the bottom of the joint to the top. Because the joint cannot twist about the inextensible spine (to which the *z* axis is tangent) the axis-angle vector consists of only two non-zero variables which we call *u* and *v*. These values are labeled in Figure 1 and correspond to the rotation about the *x* and *y* axes, respectively. We assume that the spine is perfectly inextensible so that *h* in this work becomes a constant kinematic parameter.

**Figure 2:**
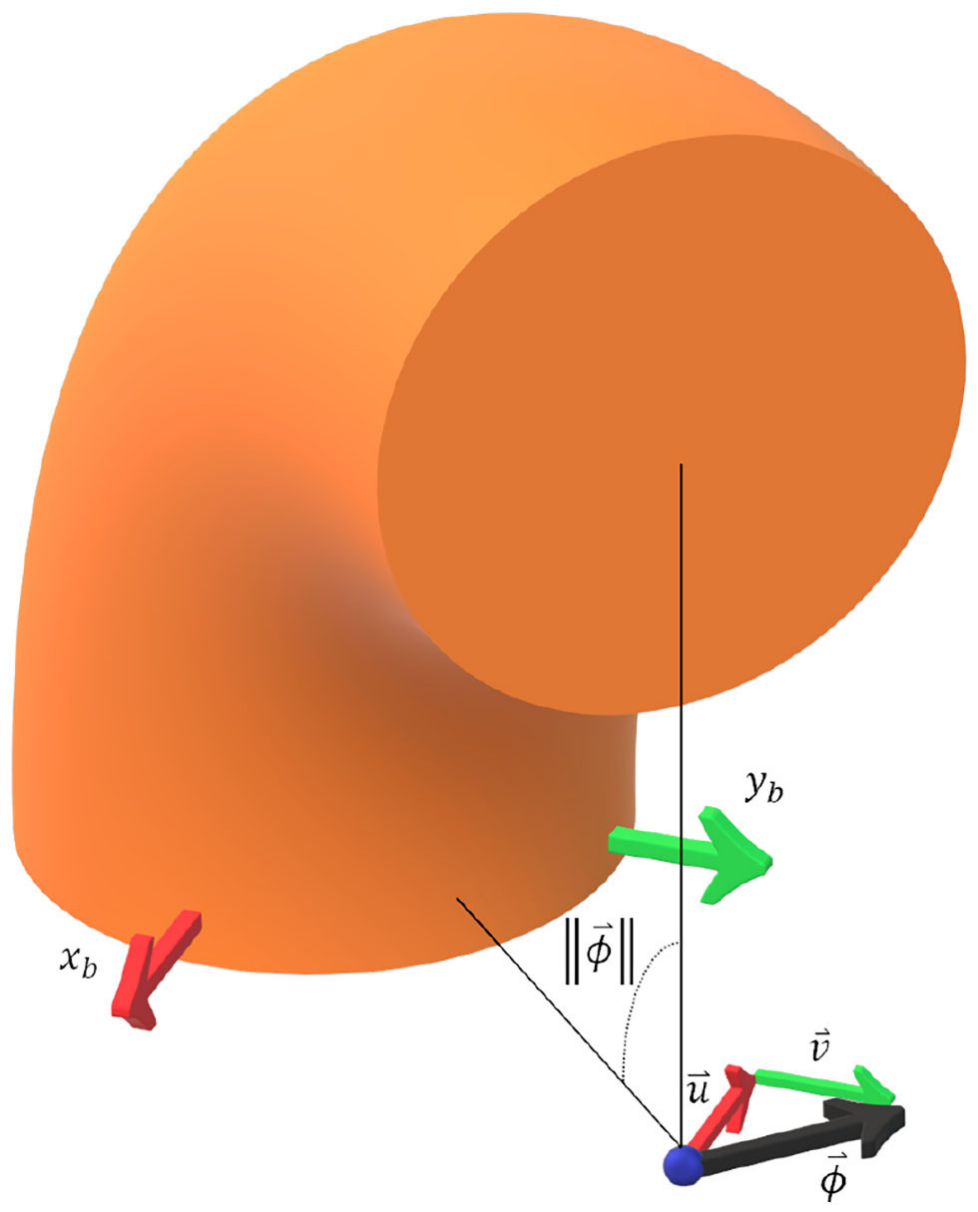
A 3D schematic to illustrate the kinematic relationships used in the presented model derivation. $\vec{\phi }$ is the axis-angle vector which can be decomposed into components parallel with the base frame b. Note that $\vec{u}$ points in the negative *x*_*b*_ direction and that $\vec{v}$ points in the positive *y*_*b*_ direction. The magnitude of the axis-angle vector $\vec{\phi }$ is also the total bend angle.

First we note some useful kinematic relationships. Because *u* and *v* are the non-zero elements of the axis-angle vector we can write

(1) $$\begin{array}{l} \phi =\sqrt{u^{2}+v^{2}} \end{array}$$

where ϕ is the magnitude of the axis-angle vector [*u, v*, 0]^*T*^, or total bend angle (see Figure 2).

**Figure 3:**
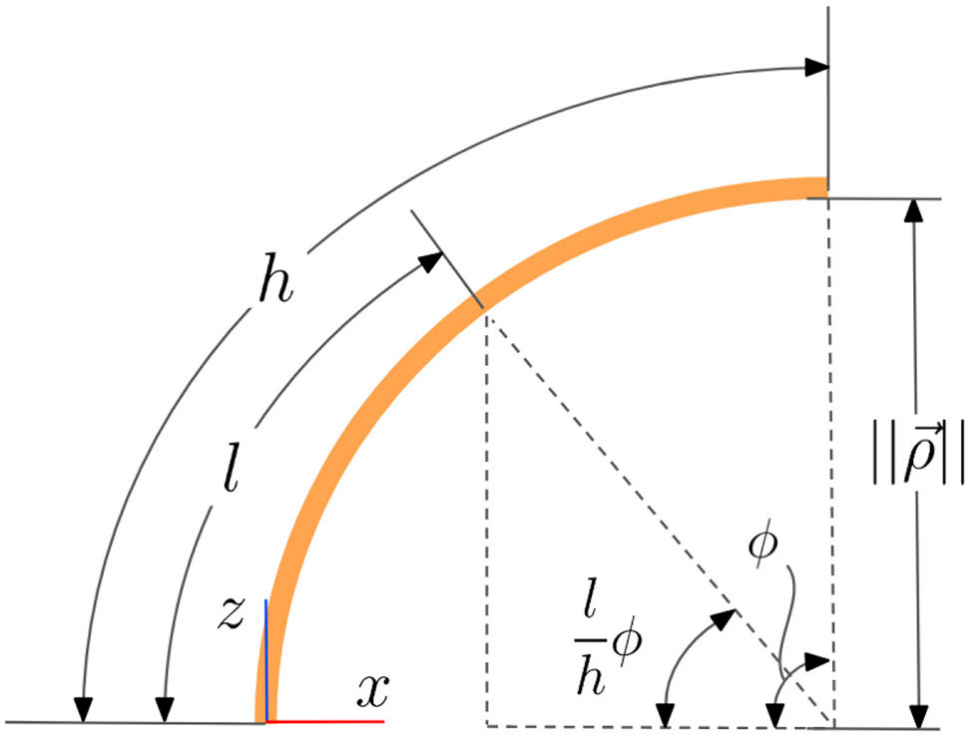
A side view of the kinematic model of a continuum joint showing trigonometric relationships between variables.

Although the joint is modeled as an arc with an arc length of *h*, we often want to refer to a position at some intermediate point along the arc. We denote an intermediate length along the arc using the variable *l* where *l* can take on any value between 0 and *h* (see Figure 3). Note that a frame tangent to the arc at a length *l* rotates as *l* is increased, therefore ϕ_*l*_, *u*_*l*_, and *v*_*l*_ are not constant along the entire arc. However, we note that the vector $\vec{\rho }$ from the base of the joint to the center of curvature is the same for all points along the arc because the center of curvature does not move. At any point *l* along the arc this vector can be calculated as

(2) $$\begin{array}{l} \vec{\rho }=\frac{l}{\phi_{l}^{2}}\left[\begin{array}{c} v_{l}\\ -u_{l}\\ 0 \end{array}\right]. \end{array}$$

Because the magnitude of this vector $\left\|\vec{\rho }\right\|$ is the radius of curvature, we may also relate ϕ and *l* using the arc-length formula

(3) $$\begin{array}{l} \phi_{l}=\frac{l}{\left\|\vec{\rho }\right\|} \end{array}$$

We now wish to derive a means by which we can calculate *u*_*l*_ and *v*_*l*_ at any point *l* along the arc given only *l*, *h*, and *u* and *v* at the end of the arc. Given a point that lies at a distance *l* along the arc, we may say using Equation (2)

(4) $$\begin{array}{l} \;{\vec{\rho }}_{l}={\vec{\rho }}_{h}\\ \frac{l}{\phi_{l}^{2}}\left[\begin{array}{c} v_{l}\\ -u_{l}\\ 0 \end{array}\right]=\frac{h}{\phi_{h}^{2}}\left[\begin{array}{c} v_{h}\\ -u_{h}\\ 0 \end{array}\right]. \end{array}$$

Replacing ϕ terms using Equation (3) we obtain

(5) $$\begin{array}{l} \frac{l\|\vec{\rho }{\|}^{2}}{l^{2}}\left[\begin{array}{c} v_{l}\\ -u_{l}\\ 0 \end{array}\right]=\frac{h\|\vec{\rho }{\|}^{2}}{h^{2}}\left[\begin{array}{c} v_{h}\\ -u_{h}\\ 0 \end{array}\right]\\ \;\left[\begin{array}{c} v_{l}\\ u_{l}\\ 0 \end{array}\right]=\frac{l}{h}\left[\begin{array}{c} v_{h}\\ u_{h}\\ 0 \end{array}\right]. \end{array}$$

Differentiating with respect to time yields the relationship

(6) $$\begin{array}{l} \left[\begin{array}{c} {\dot{v}}_{l}\\ {\dot{u}}_{l}\\ 0 \end{array}\right]=\frac{l}{h}\left[\begin{array}{c} {\dot{v}}_{h}\\ {\dot{u}}_{h}\\ 0 \end{array}\right] \end{array}$$

In other words, the generalized coordinates *u*_*l*_ and *v*_*l*_ and their time derivatives vary linearly along the length of the arc. This becomes a very useful property of this kinematic representation when deriving equations of motion.

Using the method of Lagrange, the equations of motion for a system of rigid bodies take the form

(7) $$\begin{array}{l} M\left(q\right)\ddot{q}+C\left(\dot{q},q\right)\dot{q}+g\left(q\right)=\tau \end{array}$$

where *M*(*q*) is the mass matrix, $C\left(\dot{q},q\right)$ is the Coriolis matrix, *g*(*q*) is a vector of gravity torques, *q* is a vector of the generalized coordinates, and τ is a vector of the generalized torques including friction terms. These matrices are derived using partial derivatives of kinetic and potential energy terms. Since partial derivatives are easily taken using a symbolic mathematics toolbox such as Sympy (see Meurer et al., 2017), the problem of dynamic modeling is reduced to the selection of generalized coordinates and the representation of kinetic and potential energy.

In order to accurately express kinetic and potential energy we choose to model the continuum joint, as many have done before, with an infinite set of infinitesimally small disks. However, the assumption of constant curvature, the choice of generalized coordinates, and current tools in symbolic math libraries allows us to produce analytical expressions for *M*, *C*, and *g*, whereas previous methods have not yielded these closed-form expressions.

We can define the kinetic energy of an infinitesimally thin disc at a length *l* along the arc as

(8) $$\begin{array}{l} T_{l}=\frac{1}{2}\left(\mu \text{d}l\right){\dot{p}}_{l}^{T}{\dot{p}}_{l}+\frac{1}{2}\omega_{l}^{T}I\omega_{l}\\ \;=\frac{1}{2}\left(\mu \text{d}l\right){\dot{p}}_{l}^{T}{\dot{p}}_{l}+\frac{1}{2}\omega_{l}^{T}\left[\begin{array}{ccc} \frac{\mu \text{d}lr^{2}}{4} & 0 & 0\\ 0 & \frac{\mu \text{d}lr^{2}}{4} & 0\\ 0 & 0 & \frac{\mu \text{d}lr^{2}}{2} \end{array}\right]\omega_{l}\\ \;=\frac{1}{2}\left(\mu \text{d}l\right){\dot{p}}_{l}^{T}{\dot{p}}_{l}+\frac{1}{2}[\frac{\mu \text{d}lr^{2}}{4}\omega_{l,x}^{2}+\frac{\mu \text{d}lr^{2}}{4}\omega_{l,y}^{2}+\frac{\mu \text{d}lr^{2}}{2}\omega_{l,z}^{2}]\\ \;=\frac{\mu }{2}[{\dot{p}}_{l}^{T}{\dot{p}}_{l}+r^{2}(\frac{1}{4}\omega_{l,x}^{2}+\frac{1}{4}\omega_{l,y}^{2}+\frac{1}{2}\omega_{l,z}^{2})]\text{d}l \end{array}$$

where μ is the linear density of the disc, d*l* is some infinitesimal length, $\dot{p}$_*l*_ is the velocity of the center of the disc, ω_*l*_ is the angular velocity of the disc expressed in the disc frame, and *I* is the inertia of the infinitesimally thin disc expressed in the disc frame.

The linear and angular velocity of each disc ($\dot{p}$_*l*_ and ω_*l*_) can be found using a configuration dependent jacobian *J* (meaning it is a function of joint configuration variables *u*_*l*_ and *v*_*l*_) that is defined such that

(9) $$\begin{array}{l} \left[\begin{array}{c} {\dot{p}}_{l}\\ \omega_{l} \end{array}\right]=J\left(u_{l},v_{l},l\right)\left[\begin{array}{c} \dot{u_{l}}\\ \dot{v_{l}} \end{array}\right]\\ \left[\begin{array}{c} \dot{p_{l}}\\ \omega_{l} \end{array}\right]=\left[\begin{array}{c} J_{{\dot{p}}_{l}}\left(u_{l},v_{l},l\right)\\ J_{\omega_{l}}\left(u_{l},v_{l},l\right) \end{array}\right]\left[\begin{array}{c} \dot{u_{l}}\\ \dot{v_{l}} \end{array}\right]\\ \left[\begin{array}{c} \dot{p_{l}}\\ \omega_{l} \end{array}\right]=\left[\begin{array}{c} J_{{\dot{p}}_{l}}\left(u_{l},v_{l},l\right)\\ J_{\omega_{l}}\left(u_{l},v_{l},l\right) \end{array}\right]\left[\begin{array}{c} \dot{u_{h}}\\ \dot{v_{h}} \end{array}\right]\frac{l}{h}. \end{array}$$

A definition of this Jacobian for the choice of *u* and *v* as generalized coordinates can be found in Allen et al. (2020).

Using this relationship, we see that we can simplify the expression for kinetic energy (Equation 8) by scaling portions of the jacobian. The new inertia-weighted jacobian is defined as

(10) $$\begin{array}{l} J_{\text{weighted}}\left(u_{l},v_{l},l\right)=\left[\begin{array}{c} \sqrt{\mu }J_{{\dot{p}}_{l,x}}\\ \sqrt{\mu }J_{{\dot{p}}_{l,y}}\\ \sqrt{\mu }J_{{\dot{p}}_{l,z}}\\ \frac{\sqrt{\mu }r}{2}J_{\omega_{l,x}}\\ \frac{\sqrt{\mu }r}{2}J_{\omega_{l,y}}\\ \frac{\sqrt{\mu }r}{\sqrt{2}}J_{\omega_{l,z}} \end{array}\right] \end{array}$$

allowing us to rewrite Equation (8) for the kinetic energy of a disc as

(11) $$\begin{array}{l} T_{l}=\frac{1}{2}{\dot{q}}^{T}J_{\text{weighted}}{\left(u_{l},v_{l},l\right)}^{T}J_{\text{weighted}}\left(u_{l},v_{l},l\right)\dot{q}\text{d}l. \end{array}$$

By treating a continuum joint as a series of infinitesimal disks and integrating the kinetic energy of each disc along the length of the arc we can write the total kinetic energy of a joint as

(12) $$\begin{array}{l} T=\frac{1}{2}{\dot{q}}^{T}[\int_{0}^{h}J_{\text{weighted}}{\left(u_{l},v_{l},l\right)}^{T}J_{\text{weighted}}\left(u_{l},v_{l},l\right)\text{d}l]\dot{q} \end{array}$$

We note here that the Jacobian can be expressed analytically at every point along the joint as a function of *l* and the configuration variables *u*_*l*_ and *v*_*l*_ (which are $\frac{l}{h}u_{h}$ and $\frac{l}{h}v_{h}$, respectively) thanks to Equation (5). Given this analytical expression for *J*_weighted_ we can integrate this expression with respect to *l* over the definite bounds 0 to *h* to get an analytical expression for $J_{\text{weighted}}^{T}J_{\text{weighted}}$, which we recognize as the joint space inertia matrix or mass matrix *M*.

We use the symbolic mathematics library Sympy (see Meurer et al., 2017) to calculate $J_{\text{weighted}}^{T}J_{\text{weighted}}$, and to integrate this expression analytically between the definite bounds 0 and *h* in order to obtain *M*(*q*). Once *M*(*q*) has been obtained symbolically, it is then relatively straightforward to take partial derivatives using Sympy in order to obtain an expression for the Coriolis matrix $C\left(\dot{q},q\right)$ from Equation (7) using the method outlined in Bruno et al. (2010). The resulting coefficients that multiply $\dot{q}$ to calculate the Coriolis matrix are commonly called the Christmases symbols of the first kind.

In order to find the gravity torques (*g*) we must first find the vector from the joint base to the joint center of mass ($\vec{p}$). By inspection we can see that a joint's center of mass must project down onto the vector $\vec{\rho }$ which is from the center of curvature to the base of the joint, however the vector to the center of mass must also contain some component in the *z* direction (orthogonal to the plane of the bottom plate of the joint). We find the components of the center of mass vector $\vec{p}$ by again dividing the joint into a series of infinitesimal disks of height d*l*.

Using the definition of the center of mass assuming the joint has uniform density along its length, the portion of $\vec{p}$ along the *z* axis is given by

(13) $$\begin{array}{l} \overset{-}{z}=\frac{\int_{0}^{h}z\text{d}V}{\int_{0}^{h}\text{d}V} \end{array}$$

Using the trigonometric relationship seen in Figure 3, namely

(14) $$\begin{array}{l} z\left(l\right)=\|\vec{\rho }\|\text{sin}\left(\frac{l}{h}\phi \right) \end{array}$$

as well as the volume formula for an infinitesimally thin disc

(15) $$\begin{array}{l} \text{d}V=\pi r^{2}\text{d}l, \end{array}$$

we can now integrate to find $\overset{-}{z}$:

(16) $$\begin{array}{l} \overset{-}{z}=\frac{\int_{0}^{h}\|\vec{\rho }\|\text{sin}\left(\frac{l}{h}\phi \right)\pi r^{2}\text{d}l}{\int_{0}^{h}\pi r^{2}\text{d}l}\\ \overset{-}{z}=\frac{\pi r^{2}\|\vec{\rho }\|\int_{0}^{h}\text{sin}\left(\frac{l}{h}\phi \right)\text{d}l}{\pi r^{2}h}\\ \overset{-}{z}=\frac{-[\|\vec{\rho }\|\frac{h}{\phi }\text{cos}\left(\frac{l}{h}\phi \right){]}_{0}^{h}}{h}\\ \overset{-}{z}=\frac{-\|\vec{\rho }\|}{\phi }\left(\text{cos}\left(\phi \right)-1\right). \end{array}$$

Recognizing that $\|\vec{\rho }\|=\frac{h}{\phi }$,

(17) $$\begin{array}{l} \overset{-}{z}=\frac{h}{\phi^{2}}\left(1-\text{cos}\left(\phi \right)\right). \end{array}$$

In order to find the component of $\vec{p}$ that lies in the plane of *u* and *v* we follow a similar procedure. We will use *x* to represent the portion of $\vec{p}$ that lies along $\vec{\rho }$. Using the trigonometric relationship seen in Figure 3, namely

(18) $$\begin{array}{l} x\left(l\right)=\|\vec{\rho }\|\left(1-\text{cos}\left(\frac{l}{h}\phi \right)\right), \end{array}$$

we can now integrate to find $\overset{-}{x}$:

(19) $$\begin{array}{l} \overset{-}{x}=\frac{\int_{0}^{h}\|\vec{\rho }\|\left(1-\text{cos}\left(\frac{l}{h}\phi \right)\right)\pi r^{2}\text{d}l}{\int_{0}^{h}\pi r^{2}\text{d}l}\\ \overset{-}{x}=\frac{\pi r^{2}\|\vec{\rho }\|\int_{0}^{h}\left(1-\text{cos}\left(\frac{l}{h}\phi \right)\right)\text{d}l}{\pi r^{2}h}\\ \overset{-}{x}=\frac{\|\vec{\rho }\|[l-\frac{h}{\phi }\text{sin}\left(\frac{l}{h}\phi \right){]}_{0}^{h}}{h}\\ \overset{-}{x}=\frac{\left\|\vec{\rho }\right\|}{\phi }\left(\phi -\text{sin}\left(\phi \right)\right). \end{array}$$

Recognizing that $\|\vec{\rho }\|=\frac{h}{\phi }$,

(20) $$\begin{array}{l} \overset{-}{x}=\frac{h}{\phi^{2}}\left(\phi -\text{sin}\left(\phi \right)\right). \end{array}$$

Using the derived equations for $\overset{-}{z}$, $\overset{-}{x}$, and the normalized version of $\vec{\rho }$ we obtain the vector from the base of the joint to the center of mass:

(21) $$\begin{array}{l} \vec{p}=\frac{h}{\phi^{2}}\left[\begin{array}{c} \left(\phi -\text{sin}\left(\phi \right)\right)\frac{v}{\phi }\\ \left(\phi -\text{sin}\left(\phi \right)\right)\frac{-u}{\phi }\\ \left(1-\text{cos}\left(\phi \right)\right) \end{array}\right]. \end{array}$$

The potential energy of the joint due to gravity is simply the dot product of this vector, expressed in the inertial frame, with the gravity vector ($\vec{G}$) expressed in the same frame:

(22) $$\begin{array}{l} V=\vec{p}\cdot \vec{G}. \end{array}$$

Having calculated the potential energy due to gravity, the gravity torques are calculated simply by taking the negative partial derivative of *V* with respect to *q*:

(23) $$\begin{array}{l} g=-\frac{\partial V}{\partial q}. \end{array}$$

The method above has yielded analytical expressions for *M*, *C*, and *g* with the generalized coordinates *u* and *v*. Although complex, these closed-form expressions can be exported from the Sympy library into C code that can be evaluated within microseconds, allowing for real-time model-based control of these continuum joints.

In the absence of applied pressures, the joints used for this paper tend to drive themselves toward an equilibrium position at roughly *u* = *v* = 0 with slight overshoot and brief oscillation. This spring force could have been modeled as a part of the potential energy, however we choose to model the spring and damping separately from the traditional Lagrangian equations of motion. We approximate the spring forces as a linear spring term *K*_spring_*q* and friction as a linear viscous damping term $K_{\text{d}}\dot{q}$. Including these terms, the final model used is,

(24) $$\begin{array}{l} M\left(q\right)\ddot{q}+C\left(\dot{q},q\right)\dot{q}+g\left(q\right)=\tau -K_{\text{d}}\dot{q}-K_{\text{spring}}q \end{array}$$

### 2.2. Development of Model Reference Predictive Adaptive Control

In this section we give brief overviews of both MPC and MRAC in order to clarify notation and establish a background for the development of MRPAC. For in-depth explanations of MPC and MRAC we refer the interested reader to Hyatt et al. (2020) and Lavretsky and Wise (2013) respectively.

#### 2.2.1. Model Predictive Control

Any dynamic system may be represented in state variable form as

(25) $$\dot{\text{x}}=\text{A}(\text{x},\text{u})\text{x}+\text{B}(\text{x},\text{u})\text{u}+\text{w}(\text{x},\text{u})$$

where **x** is the vector of states, **u** is the vector of system inputs, and **w** is a vector of offsets or disturbances. By linearizing this system and using any discretization method (Euler, semi-implicit Euler, matrix exponential, etc.) we can create a linear discretized state space model:

(26) $${\text{x}}_{\text{k}+1}={\text{A}}_{\text{d}}{\text{x}}_{\text{k}}+{\text{B}}_{\text{d}}{\text{u}}_{\text{k}}+{\text{w}}_{\text{d}}.$$

The above equation can be used to forward simulate the states of our system, given initial conditions and inputs. In MPC these discretized dynamic equations are the constraints of our optimization while **x_k_** and **u_k_** are the optimization variables. In an MPC solver predicting over a horizon of *T* time steps, a trajectory optimization may be formulated as:

(27) $$\begin{array}{l} J(\text{x},\text{u})=\sum_{k=0}^{T}[{\left({\text{x}}_{goal}-{\text{x}}_{\text{k}}\right)}^{T}Q({\text{x}}_{goal}-{\text{x}}_{\text{k}})\\ \;+{\left({\text{u}}_{goal}-{\text{u}}_{\text{k}}\right)}^{T}R({\text{u}}_{goal}-{\text{u}}_{\text{k}})]\\ \;s.t.\\ \;{\text{x}}_{\text{k}+1}={\text{A}}_{\text{d}}{\text{x}}_{\text{k}}+{\text{B}}_{\text{d}}{\text{u}}_{\text{k}}+{\text{w}}_{\text{d}}\;\forall \;k=0,...,T-1 \end{array}$$

where *J* is the objective function value, **x_goal_** and **u_goal_** are the goal states and inputs respectively. Other constraints may easily be added to this formulation to place bounds on inputs or states. By defining a quadratic cost function and enforcing only linear dynamics constraints we have defined a convex optimization problem suitable for solution using a very fast convex solver. We choose to use the state of the art solver OSQP (from Stellato et al., 2017) for our implementation of MPC. In order to lengthen the horizon of MPC and decrease solve times we also use the input parameterization technique presented in Hyatt et al. (2020).

MPC solves the above trajectory optimization for the entire horizon of length *T*, however only the first input (**u_0_**) is applied to the system. After applying this input, the optimization is solved again using state information that is updated from sensor feedback. The discrete-time model can also be updated with a new linearization centered at the new operating point. This process is repeated with MPC only ever applying the first input, but solving over an entire horizon of value *T*. The fact that MPC re-solves the trajectory optimization problem with the most current state and model information is what leads to MPC being robust to model error as will be shown hereafter.

#### 2.2.2. Model Reference Adaptive Control

MRAC is a form of adaptive control that seeks to drive a system to behave like a reference system. Because we are interested in controlling continuum joint soft robots we specifically follow the implementation of MRAC outlined in Slotine and Li (1987) which is specific to robot manipulators. In this derivation of MRAC for manipulators, the authors take advantage of several special properties of manipulator dynamics. Firstly, they express the mass matrix, coriolis matrix, and gravity torques as being linear in certain manipulator parameters. Stated mathematically:

(28) $$\begin{array}{l} M\left(q\right)\ddot{q}+C\left(\dot{q},q\right)\dot{q}+g\left(q\right)=Y\left(\ddot{q},\dot{q},q\right)a=\tau \end{array}$$

where $Y\left(\ddot{q},\dot{q},q\right)$ is the *n*x*p* regressor and *a* is a *p*x1 vector containing the manipulator dynamic parameters which may be unknown or changing over time. In rigid body manipulators it can be shown that *a* contains the link masses, inertias, and the positions of centers of mass. Using the soft robot continuum joint dynamic model from section 2.1 to derive *M*, *C*, and *g* it can be seen by inspection that all of these terms are linear in the joint mass *m*, as well as square of the joint radius *r*^2^ and joint height *h*^2^.

In Slotine and Li (1987) the authors present a method by which joint accelerations need not be measured or estimated in order to calculate the regressor. Instead they exploit several properties of manipulator dynamics in order to rewrite the regressor as a function of joint positions (*q*), joint velocities ($\dot{q}$), reference system velocities (${\dot{q}}_{\text{ref}}$), and reference system accelerations (${\ddot{q}}_{\text{ref}}$):

(29) $$\begin{array}{l} \tau =Y\left(q,\dot{q},{\dot{q}}_{\text{ref}},{\ddot{q}}_{\text{ref}}\right)a. \end{array}$$

The reference system includes a set of differential equations that describe a system of our choosing with desirable characteristics (such as being a 2nd-order critically damped system with a desired rise time). This is useful in practice because while accurate measurements or estimates of joint accelerations are hard to obtain, the acceleration of the reference system is a calculated value that we know perfectly.

When using MRAC, we generally do not know the parameter vector *a* perfectly (especially for soft robots), so we desire to estimate it. We will denote our estimate $\hat{a}$. The adaptive parameter vector $\hat{a}$ is adapted according to the law:

(30) $$\begin{array}{l} \dot{\hat{a}}=-\Gamma^{-1}Y{\left(q,\dot{q},{\dot{q}}_{\text{ref}},{\ddot{q}}_{\text{ref}}\right)}^{T}s \end{array}$$

where

(31) $$\begin{array}{l} s=\dot{\tilde{q}}+\Lambda \tilde{q}\\ \dot{\tilde{q}}=\dot{q}-{\dot{q}}_{\text{ref}}\\ \tilde{q}=q-q_{\text{ref}}. \end{array}$$

The terms $\tilde{q}$ and $\dot{\tilde{q}}$ are the position and velocity tracking errors with respect to the response of the reference system, and so therefore *s* is a sort of weighted tracking error term. Γ can be thought of as the learning rate of the adaptive controller.

The final step in manipulator MRAC as explained in Slotine and Li (1987) guarantees that not only parameter error, but also position error will be driven to zero. In order to ensure this, the final control law for MRAC is defined as:

(32) $$\begin{array}{l} \tau =Y{\left(q,\dot{q},{\dot{q}}_{\text{ref}},{\ddot{q}}_{\text{ref}}\right)}^{T}\hat{a}-K_{\text{d}}s \end{array}$$

Note that because *s* is a weighted sum of our position and velocity tracking errors, the matrices *K*_*D*_ and Λ can be thought of as a feedback controller on position error. This feedback term, in addition to the feed-forward term from the adaptive parameters, helps to decrease steady-state position error.

In the above equations, Γ, Λ, and *K*_*D*_ are all tuning parameters used to determine how quickly the adaptive parameters can change and how quickly position error is driven to zero. In general, selecting higher values for the tuning parameters causes the adaptive parameters to change more quickly and the tracking error to decrease more quickly. However, as one may expect, increasing these values to be too high can lead to instability.

Defining $f=M\left(q\right){\ddot{q}}_{\text{ref}}+C\left(\dot{q},q\right){\dot{q}}_{\text{ref}}+g\left(q\right)+K_{\text{d}}\dot{q}+K_{\text{spring}}q$, the regressor used for the continuum joint soft robot in this work is of the form:

(33) $$\begin{array}{l} Y\left(q,\dot{q},{\dot{q}}_{\text{ref}},{\ddot{q}}_{\text{ref}}\right)=\left[\begin{array}{ccccc} \frac{\partial f}{\partial m} & \frac{\partial f}{\partial h^{2}} & \frac{\partial f}{\partial r^{2}} & \frac{\partial f}{\partial q} & \frac{\partial f}{\partial \dot{q}} \end{array}\right]. \end{array}$$

#### 2.2.3. Model Reference Predictive Adaptive Control

MRPAC combines the strengths of both MPC and MRAC to yield a model-based optimal controller that can adapt its model online, but remains robust to unmodeled disturbances. As with MPC we begin with a model of the system, however this time we explicitly model the error in our model as a torque disturbance term:

(34) $$\begin{array}{l} \dot{\text{x}}=\text{A}\text{x}+\text{B}\text{u}+\text{w}+\tau_{\text{disturbance}}. \end{array}$$

If the error in our model is simply due to incorrect estimates of the manipulator parameters, then we should be able to represent this disturbance exactly using the same regressor as MRAC, namely:

(35) $$\begin{array}{l} \tau_{\text{disturbance}}=-Y\left(q,\dot{q},{\dot{q}}_{\text{ref}},{\ddot{q}}_{\text{ref}}\right)\hat{a}. \end{array}$$

The negative sign is necessary because we adapt the parameters in $\hat{a}$ according to the MRAC adaptation law. MRAC's adaptation law is designed to estimate a torque that, when applied to the system, will “cancel out" the system's dynamics. In MRPAC we want to represent the system's dynamics instead of the torque needed to cancel them out. These two quantities are opposite in sign, hence the negative sign shown here.

It is important to note that in MRPAC we are using the regressor and adaptive parameters to represent our model error, while in MRAC they are used to represent the system dynamics in their entirety. We therefore can not expect $\hat{a}$ to contain the same values for MRAC and MRPAC. In fact, if given a perfect model, $\hat{a}$ should theoretically remain zero for MRPAC. This is because given a perfect model, MRPAC, like MPC, should track perfectly from the beginning and $\tilde{q}$ and $\dot{\tilde{q}}$ will remain zero. As one can see from the adaptive law in Equation (30), as long as these tracking errors remain zero, the adaptive parameters will not change.

Also, it is important to note that Γ and Λ are the only tuning parameters for the estimation of $\hat{a}$ in MRPAC. While in MRAC there is an error term multiplied by *K*_*D*_ in order to ensure that position error is decreased, in MPC the tracking error is decreased by virtue of the optimization that seeks to minimize error.

In order to make a fair comparison between MRAC and MRPAC we use the same regressor for both controllers.

## 3. Description of Experiments

Adaptive control techniques are useful in the case where we do not know a complete and accurate model of our system a priori. After all, if we did have a complete and accurate model then we could perfectly predict the behavior of our system for model-based control techniques. We will classify all modeling error into two categories: parameter mismatch and structure mismatch. Parameter mismatch correspond to terms, physical phenomena, or parameters in our model that we are accounting for, but whose values are uncertain or unknown. For example inertias, damping coefficients, and spring coefficients may be parameter mismatch. Structure mismatch in our model corresponds to phenomena that occur in the real system, but are not represented in our model for whatever reason. If we assume all spring and damping elements in our system are linear while they are in fact non-linear, then we do not have the ability to represent the non-linear effect of the spring and this non-linear effect is structure mismatch.

### 3.1. Simulation Experiments

In the simulation portion of the experiments, a simulation is created using the model outlined in section 2.1 and this simulated system is controlled using three different controllers. The goal of each controller is to drive the system to follow a reference trajectory generated by a reference system. The three controllers implemented are MPC, MRAC, and the MRPAC algorithm detailed in section 2.2.3.

The reference system used for these experiments can be thought of as two uncoupled, critically-damped mass-spring-damper systems each modeled by the equation:

(36) $$\begin{array}{l} m\ddot{x}+b\dot{x}+k\left(x-r\right)=0. \end{array}$$

The masses (of mass *m*) are driven by the springs to the reference positions (*r*) and the damping coefficient (*b*) is always chosen such that the system is critically damped ($b=\sqrt{4mk}$). The rise time of the reference system can be altered by varying the spring constant (*k*). We choose a rise time such that the system has settled to steady state within about 1 s.

As mentioned in the adaptive control literature, model parameter estimation and adaptive control schemes require sufficient “excitation” in order to converge or to adapt. We provide this excitation by changing the reference positions (*r*) of our system every 2 s. Reference positions are drawn from a uniform distribution bounded above and below by $-\frac{\pi }{2\sqrt{2}}$ and $\frac{\pi }{2\sqrt{2}}$. These bounds are chosen so that the resulting total bend angle ($\phi =\sqrt{u^{2}+v^{2}}$) is never greater than $\frac{\pi }{2}$ radians.

#### 3.1.1. Case 1: Perfect Regressor (Parameter Mismatch)

The first experiment performed is designed to show the performance of all three controllers in the case where the regressor can fully describe the dynamics of the system (e.g., there is no structure mismatch). The hypothesis to be tested is that given a perfect regressor (speaking in terms of form and not initial values), both MRAC and MRPAC should be able to compensate for the system's dynamics perfectly and should drive the system to follow the reference trajectory exactly. For MPC, since it cannot adapt its model we expect that increasing model error (but not adding additional unmodeled terms) will lead to increasing tracking error.

To test this hypothesis we control the same system using the three controllers outlined in section 2.2 (i.e., MPC, MRAC, and MRPAC) and provide MRAC and MRPAC each with the same regressor. Because MPC and MRPAC require a discretized model, we introduce model error in order to see the effect on their performance. The method used for introducing model error is to make our estimates of *h*, *m*, *K*_spring_, *K*_*damper*_ a scalar multiple of their simulated value. Because MRAC does not utilize a model apart from the regressor, it is invariant to model error. All adaptive parameters for MRAC and MRPAC are initialized at zero.

**Figure 4:**
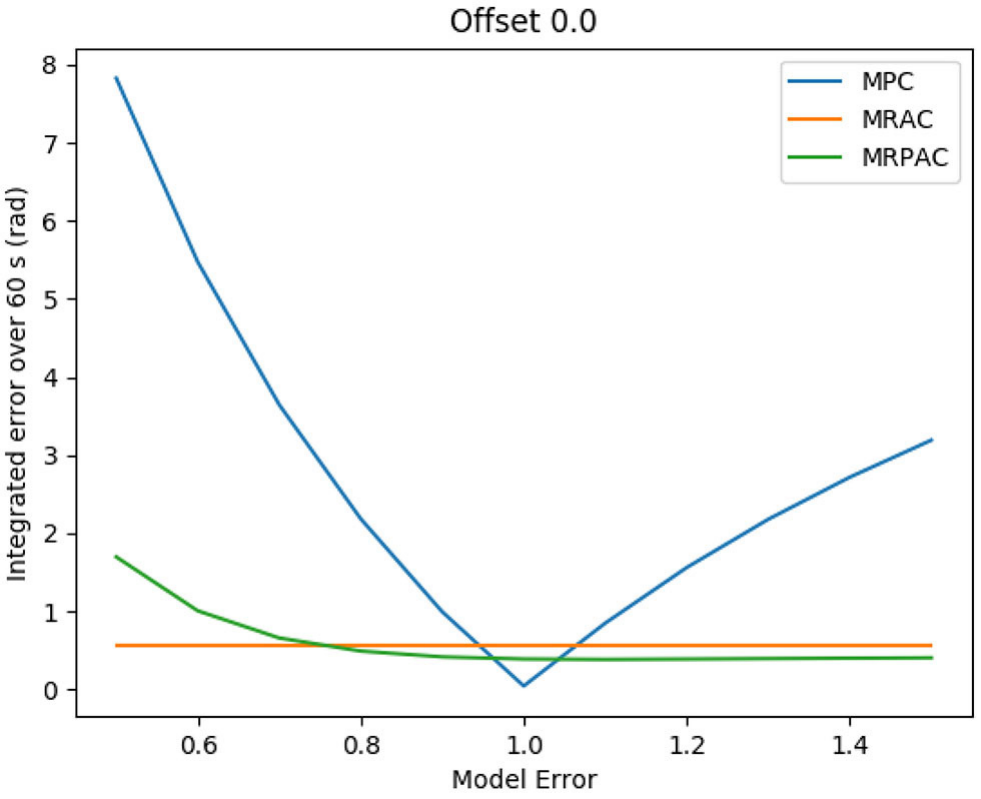
Tracking error sensitivity to model error for all three controllers in simulation.

**Figure 5:**
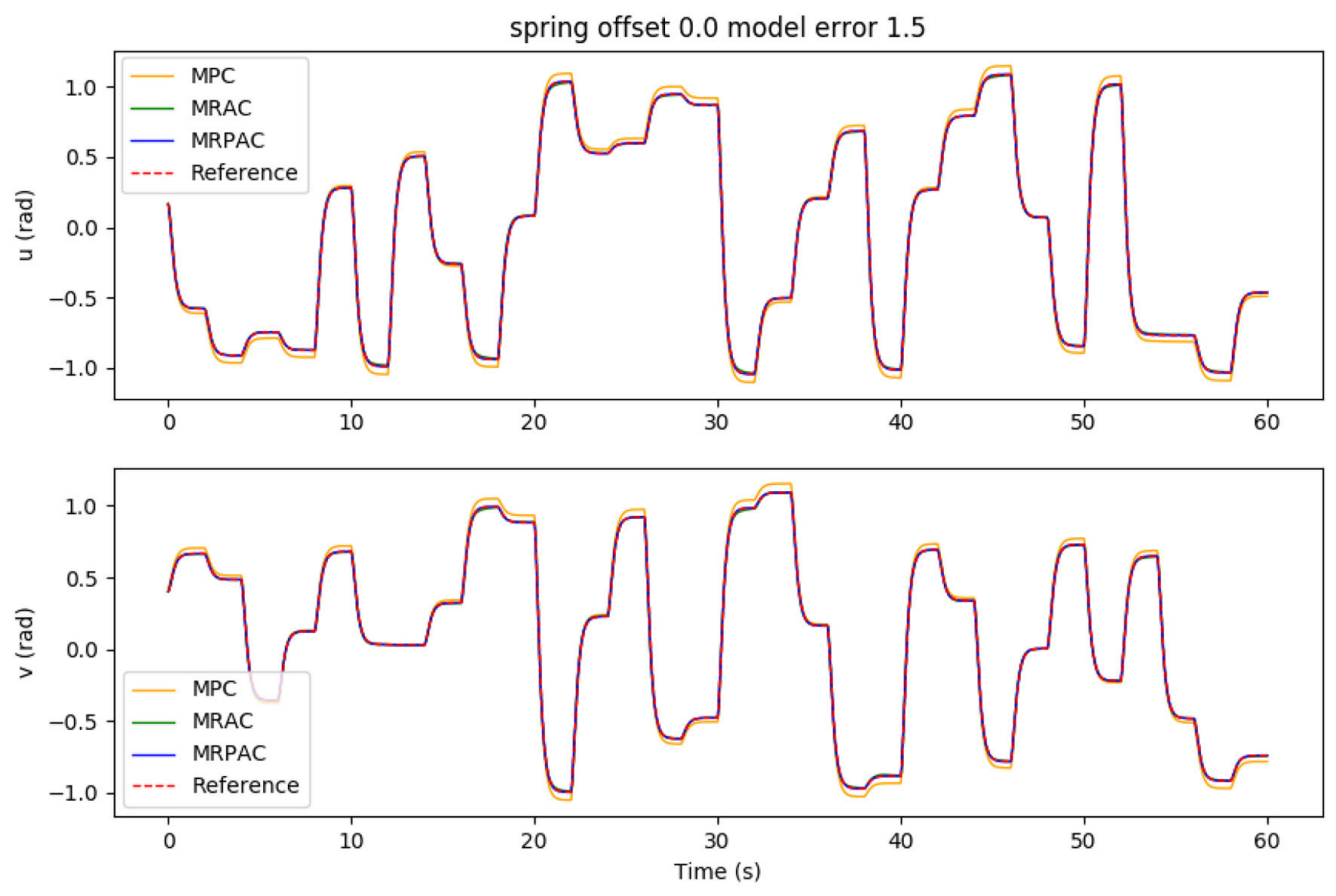
Joint trajectory tracking using all three controllers in simulation. This is for the case of errors in the model parameters used for MPC and MRPAC. Note that the reference trajectory corresponds to *q*_ref_, the position states of our dynamic reference system defined in Equation (36). Also note that the performance of MRAC and MRPAC is indistinguishable.

Each controller is run in simulation for 5 min of “excitation” (new reference commands every 2 s) in order to allow the adaptive parameters to settle. After 5 min of “excitation” the performance of each controller is evaluated during one additional minute. Because MPC is not adapting at all, this excitation period makes no difference in its performance. The integral of the position error during the 1 min evaluation is shown in Figure 4 as a function of the model error. As an example, the joint trajectories during the evaluation using a modeling error scalar of 1.5 are shown in Figure 5. Note that the green line cannot be seen because it is directly beneath the blue and red lines.

#### 3.1.2. Case 2: Imperfect Regressor (Structure Mismatch)

The second experiment performed is designed to show the performance of all three controllers in the case where the regressor cannot fully describe the dynamics of the system. The hypothesis to be tested is that neither MRAC nor MRPAC should be able to adapt for the system's dynamics perfectly given an imperfect regressor, and both should therefore struggle to drive the system to follow the reference trajectory exactly. However, because MPC has been shown to be robust to modeling error, both MPC and MRPAC should be more robust to the unmodeled forces that affect the dynamics.

To test this hypothesis, instead of simulating a system in which a spring force drives the joint toward the zero configuration, we simulate a system in which the spring force drives the joint toward a non-zero configuration. This is a phenomenon observed in real soft robot hardware because of slight inconsistencies in the manufacturing of the plastic bellows. This offset spring force can be thought of as a constant torque that is applied to the joint in one direction. Because the regressor does not contain any terms that correspond to a constant torque offset, this force cannot be represented by the regressor and therefore constitutes a “structure mismatch.” While we *do* actually know about this constant offset and likely would include a constant term in the regressor, we anticipate that there will be forces which we do not know about or whose form is unknown for any real soft robot. This simple experiment allows us to see the potential effects of these completely unmodeled forces.

In order to see the sensitivity of each controller to this unmodeled force that cannot be represented with the regressor, we vary the spring force equilibrium offset between *u* = *v* = 0.05 rad and *u* = *v* = 0.25 rad. We do this for each setting of % model error tested in the first experiment, yielding a surface of tracking error that is a function of both a scaled model error (parameter mismatch) as well as an unmodeled constant torque (structure mismatch).

**Figure 6:**
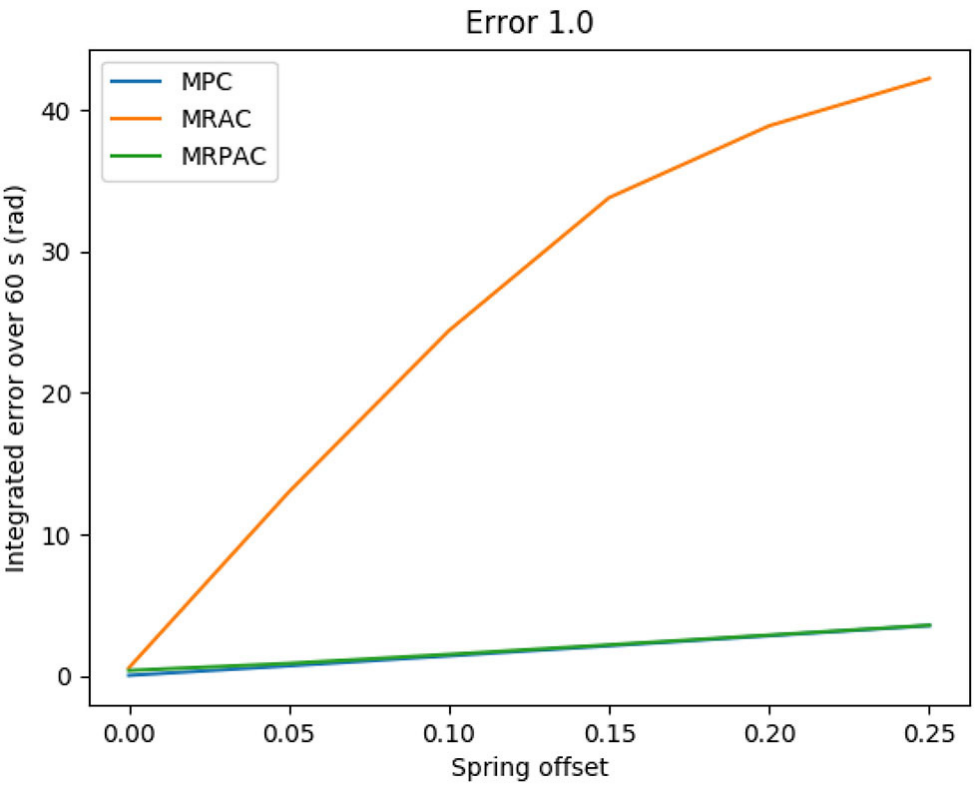
Simulated tracking error sensitivity to unmodeled offset forces/torques (structure mismatch) if the rest of the model is perfect.

**Figure 7:**
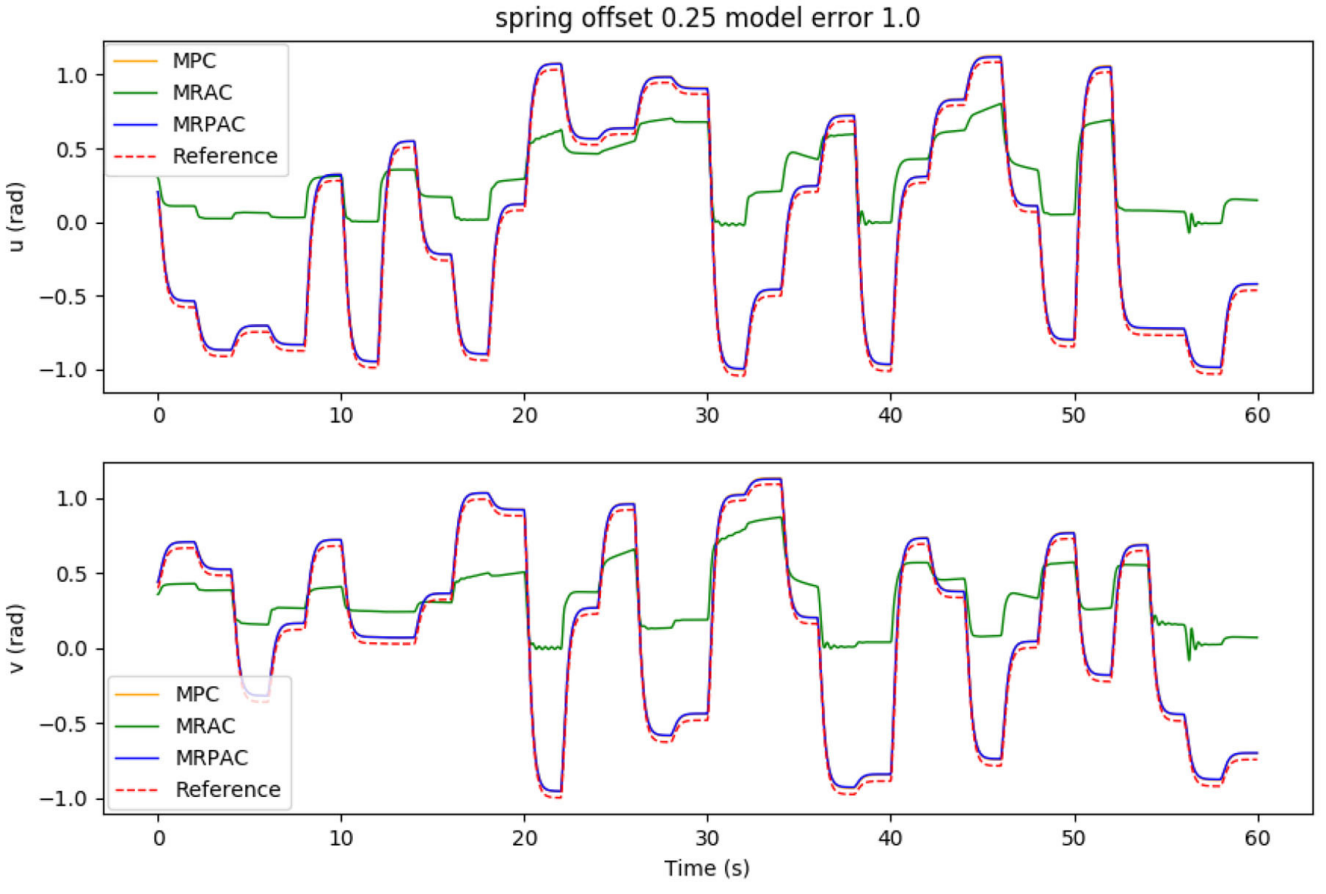
Simulated joint trajectory tracking of all three controllers with a perfect model besides an unmodeled offset torque. Note that the reference trajectory corresponds to *q*_ref_, the position states of our dynamic reference system defined in Equation (36). Also note that the performance of MPC and MRPAC is indistinguishable.

Again, after 5 min of “excitation” the performance of each controller is evaluated during one additional minute. The integrated position error during the evaluation minute is shown in Figure 6 as a function of the model error. As an example, the joint trajectories during the minute evaluation using a spring offset of *u* = *v* = 0.25 are seen in Figure 7.

### 3.2. Hardware Experiments

In order to validate both simulations, we implement the same three controllers (MPC, MRAC, and MRPAC) on the soft continuum joint shown in Figure 1 and compare their performance.

The soft continuum joint used for this experiment is actuated by four plastic bellows, each of which can be controlled independently. A pressure difference in each of the bellows causes a rotation about one or both of the joint's axes. The angle about each of these axes (denoted u and v in Figure 1) is the robot's position and are the variables that we attempt to control. We expect this hardware platform to illustrate the sensitivity of each controller to both parameter mismatch and structure mismatch.

Both sources of error are present in hardware. Because no system identification was performed previously, the aforementioned model parameters such as *h*, *m*, *r*, *K*_spring_, and *K*_d_ are not known perfectly. Additionally, the continuum joint exhibits unknown non-linear behavior near the extremes of its range of motion or in certain directions, where its stiffness or damping vary non-linearly with respect to *u* and *v*. In addition to the non-linear effects, we observe the effects of various offset forces in the plastic bellows used to actuate the joint. For example, even with equal pressures in each of the four bellows, the continuum joint remains slightly bent, indicating the presence of some constant unmodeled forces. For our simulations (see section 3.1.2) we represented this as a constant spring offset, but the actual source of this offset is unknown. In order to allow the adaptive control methods to compensate for this constant offset force we add to the regressor an identity matrix. This identity matrix means that the adaptive parameters that multiply it will be mapped directly to generalized torques in the dynamic model.

We track the orientation of a frame on top of the joint relative to a frame below the joint in order to estimate the state of the joint in real-time. We reuse the same reference trajectory from the simulation with one minor change: the command changes every 5 s instead of every two. This was adjusted in an attempt to be conservative with experimental hardware and software while still validating the performance of each controller.

As in the simulation experiments, we excite the system with the same 150 commands used in simulation (12.5 min) before evaluating each of the controllers for the last 30 commands (2.5 min). The joint trajectories for this evaluation period are shown in **Figure 9**.

## 4. Results

### 4.1. Simulation Experiments

#### 4.1.1. Case 1: Perfect Regressor (Parameter Mismatch)

The first experiment was designed to see the sensitivity of each controller to parameter mismatch, or model error where at least the form of the model is known. The results of this experiment can be seen in Figure 4. An example of the joint angle trajectories achieved by each controller is shown in Figure 5. As expected, MRAC is unaffected by this kind of model error because MRAC was initialized with all parameters equal to zero and adapted the parameters to their values based on the MRAC adaptation law. We see that given a correct form of the model, MRAC is able to find a very good model and track the reference trajectory with very little error. When MPC is given a perfect model, we see that it performs better than either MRAC or MRPAC, reducing tracking error to near zero over the entire evaluation period of 60 s. However, we see that it is the most sensitive to model error, especially when inertial, damping, and spring effects are underestimated.

The data presented in Figure 4 seem to validate the hypothesis that MRAC and MRPAC can both compensate for model error, given a model with the perfect form. We see that MRPAC is able to perform almost identically to MRAC in all cases except when inertial, damping, and spring effects are grossly underestimated. Upon further inspection of the data we found that for this case the adaptive parameters for MRPAC had not quite settled during the 5 min excitation period and that given more time, the tracking performance of MRPAC again approached that of MRAC. This is an interesting and important note - that where MPC performs worst, MRPAC has the most tracking error to overcome, and therefore may take longer to converge its adaptive parameters to a steady state. This suggests that the tuning of Γ and Λ as well as the transient response of the adaptive terms of these controllers are important topics of future research.

#### 4.1.2. Case 2: Imperfect Regressor (Structure Mismatch)

The second experiment was designed to see the sensitivity of each controller to structure mismatch, or model error where the form of the model is not known. The results of this experiment can be seen in Figure 6. An example of the joint angle trajectories achieved by each controller is shown in Figure 7. As can be seen from the figure, every controller's performance suffers because of this additional modeling error, however MRAC is by far the most sensitive. Note that the x axis of the plot denotes the value of both *u* and *v*, and the entire bend angle is equal to $\phi =\sqrt{u^{2}+v^{2}}$. Keeping this in mind, with a spring offset of about 4° (*u* = *v* = 0.05 radians) MRAC's tracking performance is worse than MPC with 50% error on estimates of masses, lengths and spring and damper coefficients. This represents a very significant decrease in performance due to a relatively small, but completely unmodeled, disturbance. This is the main motivation behind the development of MRPAC. MRPAC can be seen from this figure to inherit from MPC insensitivity to completely unmodeled disturbances or dynamics, and can be seen from Figure 4 to inherit from MRAC insensitivity to partially modeled disturbances or dynamics.

**Figure 8:**
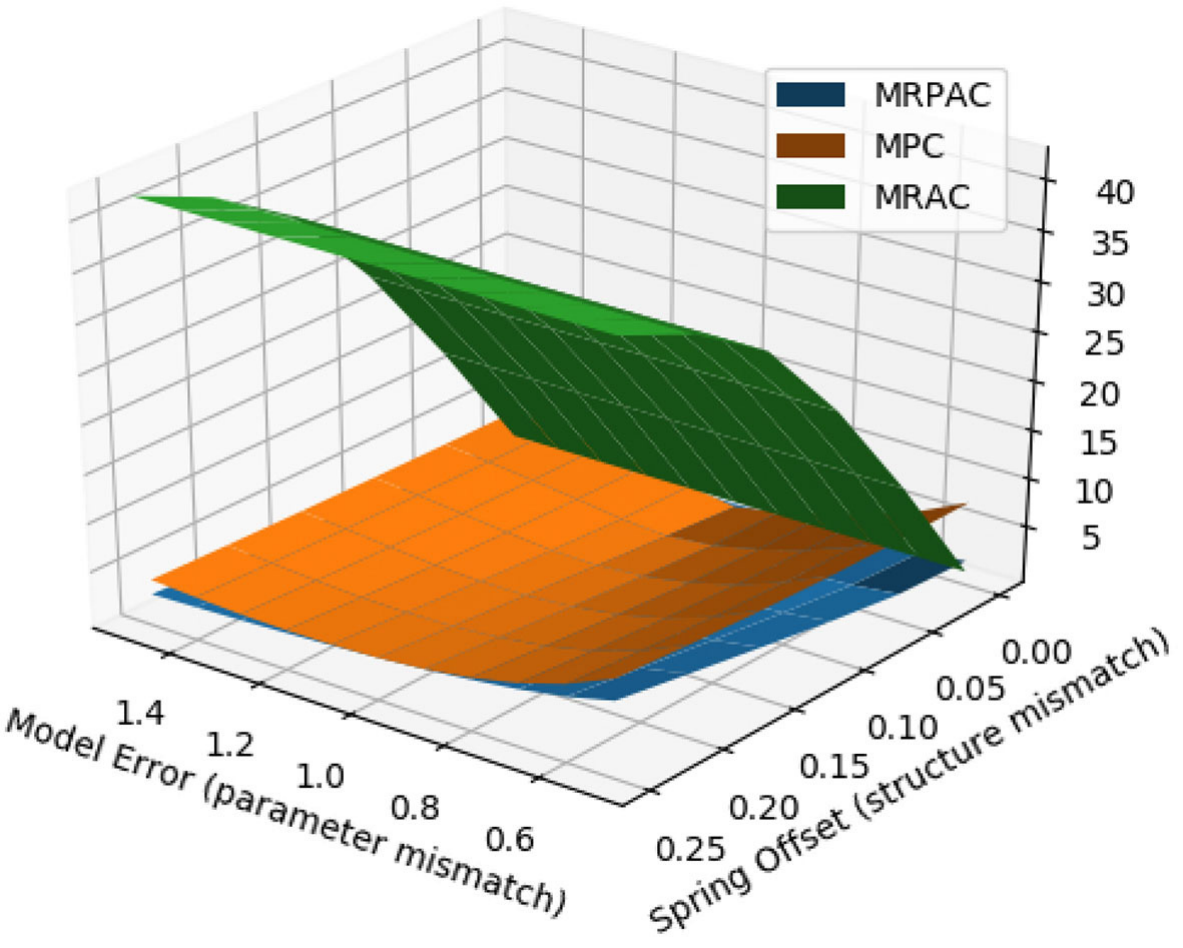
Simulated joint trajectory tracking error as a function of both model parameter error (parameter mismatch) and a spring offset error (structure mismatch).

We can vary the magnitude of both scalar modeling error as well as the unmodeled spring offset in order to develop a surface of tracking error that is a function of both parameter mismatch and structure mismatch. This surface can be seen in Figure 8. This is useful information because in reality we are likely to encounter both types of unknowns instead of just one. From the figure we can see that MRPAC consistently has the lowest tracking error of the three controllers, except when MPC has a perfect model or when the model used for MRPAC grossly underestimates inertial, damping, and spring effects. As stated earlier, we have observed that the performance of MRPAC can be improved in the latter case by allowing it to adapt for longer. However, these experimental results outline an important fact, which is that the transient responses of the adaptive terms of MRAC and MRPAC are not the same for the same Γ and Λ values. The exact differences between them and the exact reasons remain for future work.

### 4.2. Hardware Experiments

**Figure 9:**
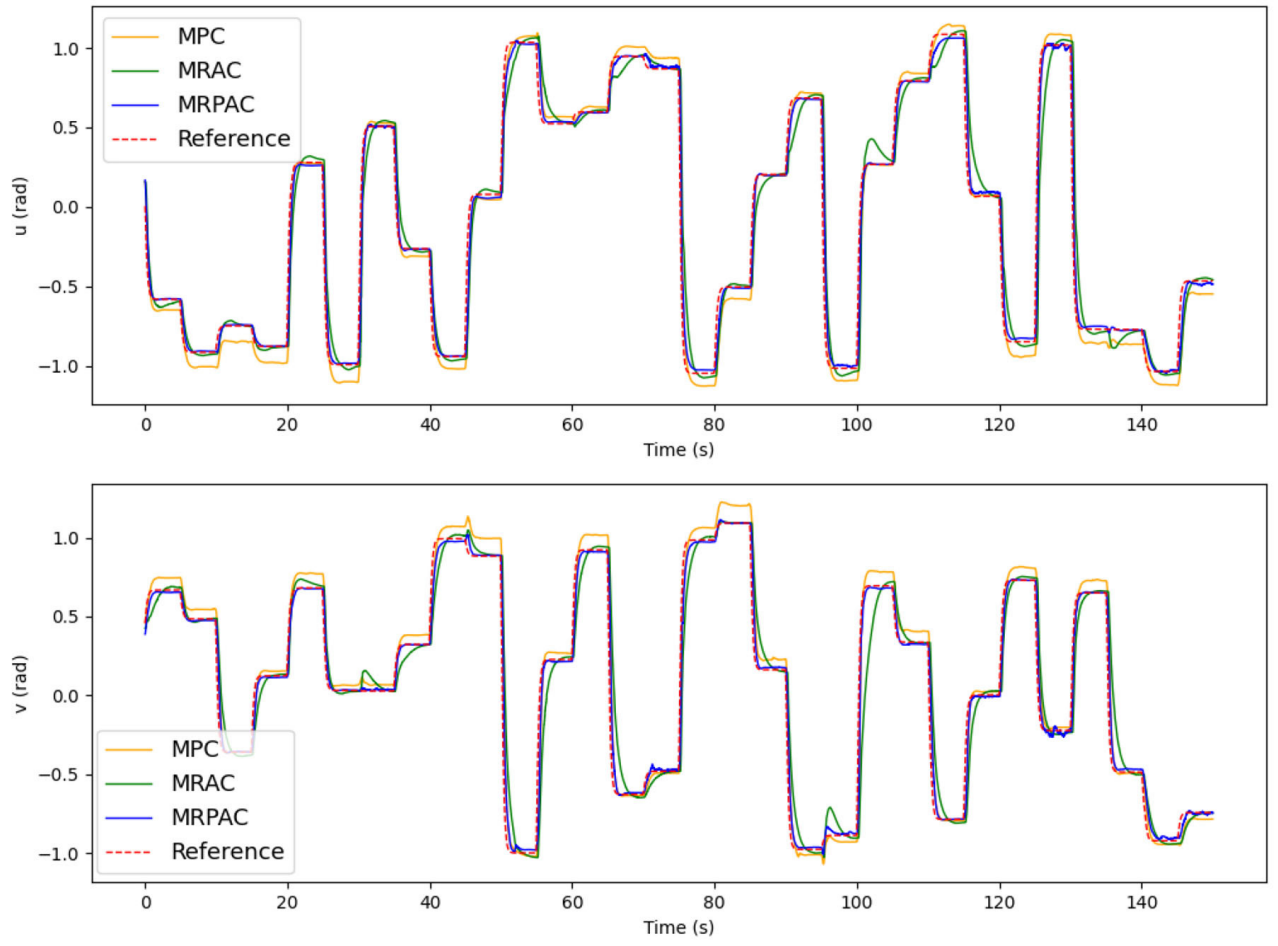
Joint trajectory tracking of all three controllers in hardware. Note that the reference trajectory corresponds to *q*_ref_, the position states of our dynamic reference system defined in Equation (36).

The joint trajectories for the hardware experiments are shown in Figure 9 and the integral of the position tracking error is reported in Table 1. It is important to note that, unlike for the simulation, we cannot separate the perfect regressor and imperfect regressor cases on real hardware. Because of the nature of the continuum joint, we expect some combination of both cases to influence the controller performance results.

**Table 1:** Position tracking error statistics for all three controllers during the 2.5 min evaluation.

	**Integrated error**	**Mean error**	**Median error**	**Std. Dev. of error**
MPC	18.24	–0.0043	–0.0037	0.1198
MRAC	21.63	–0.0027	–0.0005	0.1829
MRPAC	9.529	–0.0009	–0.0002	0.0924

Generally, we see from the results that MPC struggles to eliminate steady state error. This matches the simulated behavior in Figure 5 and is expected because MPC does not have the ability to compensate for modeling errors that exist in the continuum joint. MRAC and MRPAC, on the other hand, do have the ability to compensate for modeling errors. Consequently they both track the steady state reference trajectory much closer than MPC. This indicates that the hypothesis presented in section 3.1.1 is demonstratively true at least for this hardware platform. MRAC and MRPAC certainly compensate for the modeling errors and drive the system to follow the reference trajectory. In hardware however, we see that neither controller is capable of following the reference trajectory *exactly*. In other words, we do not see in hardware the same performance as we see in the simulation results in Figure 5, where both trajectories deviate very little from the reference. This is because in addition to the modeling error (parameter mismatch) for which MRAC and MRPAC can compensate, there are still system dynamics for which they cannot fully compensate (structure mismatch).

The effect of structure mismatch in simulation is shown in Figure 6. Tracking error increases for all control methods as the magnitude of these modeling errors increase, but they increase dramatically for MRAC, hence its poor simulation performance exhibited in Figure 7. Importantly, this same pattern emerges in our hardware experiments. There are several instances during the evaluation period where unknown forces cause deviation from the reference trajectory. For examples of this, see the upper plot (*u*) of Figure 9 at 65, 100, and 135 s and the bottom plot (*v*) at 30, 45, and 95 s. All controllers are negatively affected, but MPC and MRPAC are more robust than MRAC. In other words, when encountering such disturbances, MRAC is forced to artificially adapt dynamic parameters in an attempt to eliminate the error. In contrast, MPC and MRPAC are better able to respond to disturbances because they re-solve the trajectory optimization over the whole time horizon, not just a single time step. These results support the hypothesis outlined in section 3.1.2 as well. MRAC and MRPAC do not track the reference trajectory perfectly because of the unknown disturbances but MPC and MRPAC are quantifiably more robust to the structure mismatch.

The results reported in Table 1 add a quantitative performance analysis in addition to the qualitative analysis from Figure 9. From the table we can see that MRPAC accumulates about half of the integrated tracking error of the other two controllers during the 1 min evaluation. It is interesting to note that MPC and MRAC have similar integrated tracking error, although qualitatively their trajectories look different. While MPC has a good transient response and large steady state error, MRAC has a poor transient response and small steady state error. This is also reflected in the statistics, since MRAC has lower mean and median error than MPC, but a higher standard deviation. According to these results, it seems that MRPAC has taken the strengths of the two approaches yielding a good transient response and smaller steady state error.

## 5. Conclusions and Future Work

In this paper we have presented a novel dynamic modeling approach for one joint of a continuum joint robot. We have shown that while not linear in the same parameters as rigid robots, joint accelerations using this model can be shown to be linear in other dynamic and kinematic parameters. This linearity in model parameters can be exploited for system identification, or as we show later in the paper, for adaptive control. Future work in the area of continuum joint dynamic modeling may include system identification on hardware, as well as verification that the proposed model accurately describes the joint's dynamics. While the presented model is only valid for one joint, another straightforward extension to this work would be to derive dynamic models using the same ideas and assumptions (constant curvature assumptions, *u* and *v* parameterization) in order to derive a dynamic model for a robot with many joints and links.

In this paper we have also shown that MPC is an effective control strategy for maintaining robustness to unmodeled forces and/or dynamics. Medium to high fidelity models (such as the one presented in this paper) are promising as a means of reducing these unmodeled disturbances, but take time and effort to develop with possibly very small gains in performance. Even equipped with a perfect model, determining soft robot model kinematic and dynamic parameters accurately is a formidable task and these parameters may also change over time. As such, our presented control strategy, MRPAC, contributes a novel approach to overcoming these challenges by adapting the dynamic model while still leveraging the benefits of MPC.

Specifically, MRPAC inherits two invaluable traits: the adaptive capabilities of MRAC and the robustness of MPC. As a result, MRPAC outperforms both MPC and MRAC on a soft continuum joint, where both parameter mismatch (such as unknown spring and damper coefficients) and structure mismatch (such as unmodeled external forces or offsets) exist. MRPAC successfully compensates for modeling errors to eliminate steady state error while also demonstrating robustness to modeling disturbances.

Future research into MRPAC should include investigation into how to identify a minimal regressor that accurately represents a system's dynamics. Although not discussed in this work, the time taken by MRAC and MRPAC to converge to steady-state adaptive parameters was notably different. For MRPAC it depended heavily on the initial model parameters. The exact differences between the transient response of each control method as well as investigation into the reasons for these differences is left to future work. While our approach has shown promising results, we also did not compare it to other adaptive MPC formulations. Nor do we make the claim that it is the best adaptive MPC formulation. Future work should likely include a comparison between our approach and other existing methods.

Although the problems of accurate soft robot modeling and control remain interesting and unsolved problems, we believe that the dynamic model and adaptive control methods presented in this work represent an important contribution as a new approach to soft robot control.

## Data Availability Statement

The data of this article will be available upon request to the corresponding author.

## Author Contributions

PH and CJ participated in this research as partial fulfillment of their degrees from Brigham Young University. Furthermore, CJ participated in the data collection, problem formulation, and paper writing. MK participated in this research as an advising professor. All authors contributed to the article and approved the submitted version.

## Conflict of Interest

The authors declare that the research was conducted in the absence of any commercial or financial relationships that could be construed as a potential conflict of interest.
